# Combination of Collagen-Based Scaffold and Bioactive Factors Induces Adipose-Derived Mesenchymal Stem Cells Chondrogenic Differentiation *In vitro*

**DOI:** 10.3389/fphys.2017.00050

**Published:** 2017-02-02

**Authors:** Giovanna Calabrese, Stefano Forte, Rosario Gulino, Francesco Cefalì, Elisa Figallo, Lucia Salvatorelli, Eugenia T. Maniscalchi, Giuseppe Angelico, Rosalba Parenti, Massimo Gulisano, Lorenzo Memeo, Raffaella Giuffrida

**Affiliations:** ^1^Istituto Oncologico del Mediterraneo – Ricerca ViagrandeCatania, Italy; ^2^Physiology Section, Department of Biomedical and Biotechnological Sciences, University of CataniaCatania, Italy; ^3^Finceramica FaenzaFaenza, Italy; ^4^Anatomic Pathology Section, Department of Medical and Surgical Sciences and Advanced Technologies, G.F. Ingrassia, “Policlinico Vittorio Emanuele”, University of CataniaCatania, Italy; ^5^Department of Experimental Oncology, Mediterranean Institute of OncologyViagrande, Italy

**Keywords:** mesenchymal stem cells, 3D scaffolds, cartilage repair, chondrogenic differentiation, regenerative medicine

## Abstract

Recently, multipotent mesenchymal stem cells (MSCs) have attracted much attention in the field of regenerative medicine due to their ability to give rise to different cell types, including chondrocytes. Damaged articular cartilage repair is one of the most challenging issues for regenerative medicine, due to the intrinsic limited capability of cartilage to heal because of its avascular nature. While surgical approaches like chondral autografts and allografts provide symptoms and function improvement only for a short period, MSC based stimulation therapies, like microfracture surgery or autologous matrix-induced chondrogenesis demonstrate to be more effective. The use of adult chondrocytes, which are the main cellular constituent of cartilage, in medical practice, is indeed limited due to their instability in monolayer culture and difficulty to collect donor tissue (articular and nasal cartilage). The most recent cartilage engineering approaches combine cells, biomaterial scaffold and bioactive factors to promote functional tissue replacements. Many recent evidences demonstrate that scaffolds providing specific microenvironmental conditions can promote MSCs differentiation toward a functional phenotype. In the present work, the chondrogenic potential of a new Collagen I based 3D scaffold has been assessed *in vitro*, in combination with human adipose-derived MSCs which possess a higher chondrogenic potential compared to MSCs isolated from other tissues. Our data indicate that the scaffold was able to promote the early stages of chondrogenic commitment and that supplementation of specific soluble factors was able to induce the complete differentiation of MSCs in chondrocytes as demonstrated by the appearance of cartilage distinctive markers (Sox 9, Aggrecan, Matrilin-1, and Collagen II), as well as by the cartilage-specific Alcian Blue staining and by the acquisition of typical cellular morphology. Such evidences suggest that the investigated scaffold formulation could be suitable for the production of medical devices that can be beneficial in the field of articular cartilage engineering, thus improving the efficacy and durability of the current therapeutic options.

## Introduction

Chondrogenesis is the biological process leading to the formation of hyaline, fibrous, and elastic cartilage. Chondrocytes, which are the unique cellular phenotype in cartilage and differentiate after the condensation of MSCs (Stott et al., 1999; Ghosh et al., 2009), can either remain in a quiescent status to form the articular cartilage, or can proliferate, assuming a hypertrophic morphology and undergo to the endochondral ossification process. In such process, the embryonic cartilaginous model of long bones is gradually replaced by bone tissue, contributing to bone longitudinal growth (Mackie et al., 2008).

Within cartilage, chondrocytes are responsible for the secretion of extracellular matrix (ECM) molecules, such as proteoglycans, mainly Aggrecan, which form the extrafibrillar matrix and collagens, mainly Collagen II, which form the fibrillar matrix.

It is well known that damaged cartilage has poor intrinsic regenerative capacity, due to the peculiar nature of the tissue itself, lacking blood and lymphatic networks; consequently chondrocytes have a limited availability of oxygen and nutrients (Lafont, 2010; Madry et al., 2010).

The current clinical approaches aimed to repair and regenerate a damaged articular cartilage include osteochondral transplantation (Chow et al., 2004; Coons and Barber, 2005), MSC stimulation based therapies like microfracture surgery and, more recently, cell-based strategies such as autologous chondrocytes (O'Driscoll, 1998; Richardson et al., 1999) and MSCs implantation (Wakitani et al., 2011; Jo et al., 2014). The use of differentiated chondrocytes transplantation has shortly shown its limitations in the functional restoration of chondral tissue due to the scarcity of donor sites (cells can be collected only from articular and nasal cartilage) and to the instability of cells grown in monolayer culture, as chondrocytes dedifferentiate to a fibroblastic phenotype (Benya and Shaffer, 1982). On the other hand, MSC implantation seemed to be the most reliable approach for cartilage regeneration, seen recent clinical studies, utilizing the intra-articular injection of MSCs for cartilage repair, which reported a significant reduction of pain, the restoration of tissue functionality and the regeneration of hyaline-like cartilage (Wakitani et al., 2011; Jo et al., 2014). Intra-articular MSCs implantation is usually performed using biocompatible hydrogels in order to promote local cells attachment resembling the ECM microenvironment and to avoid unwanted cell loss (Spiller et al., 2011). To enhance the performance of these materials in achieving a complete restoration of functional cartilage, a number of critical issues have to be addressed. An optimal balance between physical (density, porosity, elasticity, flexibility, ability to sustain, and transmit pressure loads) and biological properties (biocompatibility, absence of cytotoxicity and antigenicity, capable of promoting cell attachment, proliferation, and differentiation) of the filling material is mandatory for an adequate clinical translation and additional issues regarding isolation and manipulation of cells still need to be addressed (Minguell et al., 2013; Hoch and Leach, 2014). The identification of companion chemical inducers and modulators that may strengthen the innate properties of the biomaterial to support and guide chondrogenesis may also be extremely beneficial and lead to the development of complete and effective therapeutic strategies in orthopedic regenerative medicine.

For these reasons the development of new technological solutions able to promote the restoration of chondral tissue structure and its functional properties represent attracting alternatives for cartilage therapy.

Recently, our laboratory showed the potential of a novel collagen/hydroxyapatite biomimetic scaffold in inducing new bone formation both *in vitro*, in combination with human MSCs (Calabrese et al., 2016a) and *in vivo*, after implantation of the empty scaffold into the dorsum of mice (Calabrese et al., 2016b). These studies demonstrated that collagen-hydroxyapatite scaffold was able to commit human MSCs toward osteogenic differentiation already *in vitro* (Calabrese et al., 2016a); in addition, the same type of scaffold, *in vivo*, was able to recruit host MSCs and to induce these cells to differentiate toward an osteocyte-like phenotype and, hence, to stimulate the formation of new bone tissue (Calabrese et al., 2016b).

In the same way, in this work, we have evaluated, *in vitro*, the chondrogenic differentiation potential of a new 3D scaffold mainly composed by equine type I Collagen, in combination with human adipose-derived MSCs which possess a higher chondrogenic potential compared to MSCs isolated from other tissues (Calabrese et al., 2015), either in absence or presence of chondrogenic inducing factors. The microstructural properties of the proposed scaffold composition resembles the chondral tissue in term of density and elasticity but its Collagen I based composition allow to couple its biocompatibility and biomimetic properties to a simple and inexpensive productive process (Deponti et al., 2014).

## Materials and methods

### Scaffold structure

Cylindrical scaffolds were manufactured by Fin-Ceramica Faenza SpA (Faenza–Ravenna, Italy) starting from equine type I collagen gel (1 wt%) supplied in aqueous acetic buffer solution (pH = 3.5) (Opocrin SpA, Modena, Italy). The collagen gel was gently diluted in highly purified water and precipitated in fibers by drop-wise addition of 0.1 M NaOH solution up to the isoelectric point (pH = 5.5). In order to stabilize the scaffold whole structure, the molecular links between fibers was chemically optimized by using 1,4-butanediol diglycidyl ether (BDDGE) as crosslinking agent. The crosslinking reaction was achieved by 48 h-long immersion of the agglomerated fibers at 37°C in NaHCO3/Na2CO3 (Sigma Aldrich and Merck Millipore) buffered aqueous solution with a BDDGE solution ratio equals to 1 wt%.

The agglomerated fibers were then freeze-dried with a controlled freezing and heating ramp from 25°C to −35°C and from −35°C to 25°C achieving a porous 3D structure. The process was carried out over a period of 25 h under vacuum conditions (*P* = 0.29 mbar). Scaffold was then reshaped in cylinders of required diameters and height for physical characterization (Ø = 10–18 mm, h = 4 mm), *in vitro* and *in vivo* analysis (Ø = 8 mm, h = 5 mm). Scaffolds were gamma-sterilized at 25 kGy.

Scaffolds were characterized in porosity, swelling behavior and material density.

The morphological and microstructural analysis was executed by Scanning Electron Microscopy (SEM) performed on a SEM-LEO 438 VP (Carl Zeiss AG, Oberkochen, Germany). The samples were sputter-coated with gold prior to examination. 3 SEM micrographs were analyzed by image J software. The mean pore diameter was calculated as average of the major and minor axes of the ellipse representing pores cross-section. A total of 327 pores were analyzed obtaining a mean value of 67 ± 31 micron.

The swelling capacity of the material was evaluated on 20 cylindrical scaffolds (ø = 10 mm, h = 4 mm) of cartilage-like composition. Dry scaffolds were soaked at room temperature in PBS (Alchimia) until stabilization of scaffold dimension was reached. The swelling was then determined as percent increase in both dimensions and evaluating the weight increase as described by Ma et al. (2003). Outlier values (Huber test) were deleted from the data analysis.

The density and porosity of the collagen-based scaffold were evaluated with a glass pycnometer full of highly purified water on 20 scaffolds (*d* = 18 mm; *h* = 4 mm) (She et al., 2007).

The porosity of the scaffolds was then evaluated using the average value of the densities obtained and the geometric volume of the scaffolds.

(1) $$\begin{array}{r} \varphi =1-\left(\text{M}1/\text{Vg}\right)/\rho \text{r} \end{array}$$

where:

M1 = Mass of collagen scaffold
Vg = Geometric Volume of the scaffold (cylinder)
ρr = mean value density
Samples have been tested in triplicate.

### Isolation, expansion and characterization of human adipose derived stem cells

Human adipose derived stem cells (hADSCs) were derived from adipose tissue biopsies/lipoaspirates supplied by Mediterranean Institute of Oncology (IOM) (Viagrande, Italy) under an approved Institutional Review Board protocol (project ID code: 829_1 of 8 February 2013, IOM Institutional Review Board) and after informed consent. Isolation from adipose tissue, expansion and characterization by flow cytometry analysis using several MSCs surface markers of hADSCs was performed as previously reported (Calabrese et al., 2015, 2016a; Vicari et al., 2016). In addition, cells were characterized also by immunocytochemistry analysis using several positive (CD105, CD90, CD73, CD271) and negative (CD45, CD34, CD31, and GlycoforinA) MSCs surface markers.

Immunocytochemistry was performed on cells seeded in 8-well BD Falcon culture slides at a density of 5000 cells per cm^2^ in MSC-GM (Lonza, Basel, Switzerland). The primary incubation was performed, overnight at 4°C, with the following anti-human antibodies: mouse CD105 (1:50, Novus Biologicals, Littleton, CO, USA), mouse CD90 (1:50, Santa Cruz Biotechnology, Dallas, TX, USA), mouse CD73 (1:25, Novus Biologicals), mouse CD271 (1:100, Santa Cruz Biotechnology), rabbit CD45 (1:100, Epitomics, Burlingame, CA, USA), rabbit CD34 (1:100, Epitomics), mouse CD31 (1:100, Santa Cruz Biotechnology), and goat polyclonal anti Glycoforin A (1:100, Novus Biologicals). After washing, slides were incubated with the appropriate secondary AlexaFluor 568 and 488 antibodies (Life Technologies Italia, Monza, Italy) at the dilution of 1:2000 for 1 h at RT. Nuclei were counterstained with DAPI (4',6-diamidino-2-phenylindole, 1:10,000). Finally, slides were mounted in fluorescent mounting medium Permafluor (Thermo Scientific, Waltham, MA, USA) and digital images were acquired using a Leica DMI4000B fluorescence microscope (Leica, Wetzlar, Germany). Control of immunostaining specificity was performed by omitting the primary antibody.

For flow cytometry analysis, cells were detached with 0.05% trypsin/EDTA and washed in PBS. 1 × 10^4^ cells/tube were stained with the following antibodies: CD45 FITC (Clone J.33), CD34 PE (Clone 581), Glycophorin A PE (Clone 11E4B-7-6), CD73 PE (Clone 581), CD90 FITC (Clone F15.42.1.5), CD105 PE (Clone 1G2), CD31PE (Clone 1F11), CD271 FITC (Clone ME20.4-1.H4) and corresponding isotypic controls according to manufacturer indications. All antibodies were purchased from Beckman Coulter (Milano,Italy), except CD271 that was provided by Miltenyi Biotec (Bologna, Italy). All tubes were incubated in the dark for 20 min at room temperature. Cells were then washed with PBS and finally analyzed by flow cytometry using an FC-500 five-color flow cytometer (Beckman Coulter, Pasadena, CA, USA). For each tube, 1000 events were acquired. CXP Analysis software (Beckman Coulter, Inc.) was used for data analysis.

### hADSC chondrogenic differentiation

hADSC chondrogenic differentiation was achieved as previously described (Calabrese et al., 2015). Briefly, 2.5 × 10^5^ cells were centrifuged to form a three-dimensional aggregate and resuspended in complete chondrogenic medium containing differentiation basal medium (Chondrogenic Basal Medium, Lonza) supplemented with chondrogenic differentiation inducing factors (hMSC Chondrogenic SingleQuots containing Dexamethasone, Ascorbate, ITS+supplement, GA-1000 Sodium Pyruvate, Proline and L-Glutamine, Lonza) and TGF-β3 (Lonza). Pellets were incubated at 37°C in a humidified atmosphere of 5% CO_2_. The growth medium was replaced every 2–3 days. The chondrogenic differentiation was completed on day 28 after induction. Pellets were fixed in formalin at three different time points along differentiation (1, 2, and 4 weeks). Subsequently, pellets were paraffin embedded and cut into 3 μm-thick sections for immunohistological processing.

### Immunohistochemical analysis of pellets

After deparaffinization and rehydration, sections were permeabilized with 0.4% Triton-X100, blocked with 4% BSA and then incubated overnight at 4°C with the following rabbit polyclonal primary antibodies: anti-Aggrecan (1:150), anti-Matrilin-1 (1:200), anti-Sox9 (1:200), anti-type II Collagen II (1:200), all purchased from LSBio (Seattle, WA, USA). The following day, sections were incubated for 1 h at RT with the Alexa Fluor anti-rabbit 568 secondary antibodies (1:2000, Life Technologies). Then slides were counterstained with DAPI (1:10,000) and mounted with Permafluor (Thermo Scientific). Control of immunostaining specificity was performed by omitting the primary antibody.

### hADSC chondrogenic differentiation on scaffolds

hADSC chondrogenic differentiation on scaffolds was performed as previously described (Calabrese et al., 2016a). Briefly, 2 × 10^6^ hADSCs at passage 3 were slowly drip seeded onto the scaffold and incubated in 24-well culture plates for 4 h at 37°C. ADSC-GM medium (2 ml) (Lonza, Basel, Switzerland) was added and 24 h later (day 0) the medium was replaced with chondrogenic or expansion medium. Both media were completely replaced twice a week. Each scaffold was analyzed on week 1, 2, 4, and 8 after chondrogenic induction.

### Histological and immunohistochemical analysis on hADSCs-scaffold

Scaffolds seeded with hADSC were fixed in 4% PFA at the different time points analyzed (1, 2, 4, and 8 weeks), dehydrated, embedded in paraffin and cut into 3 μm-thick sections. Sections were mounted on slides and processed for immunohistochemical staining as above reported, using the same rabbit polyclonal primary antibodies: anti-Matrilin 1 (1:200, LSBio), anti-Aggrecan (1:150, LSBio), anti-Sox 9 (1:200, LSBio) and anti-Collagen II (1:200, LSBio). The following day, sections were incubated for 1 h at RT with the Alexa Fluor anti-rabbit 568 secondary antibodies (1:2000, Life Technologies Italia, Monza, Italy). Then slides were counterstained with DAPI and mounted with Permafluor. Control of immunostaining specificity was performed by omitting the primary antibody. Alternate sections were also labeled with Haematoxylin and Eosin (H&E) and with Alcian Blue staining (Panreac, Castellar del Valles, Barcellona, Spain). For the staining an Alcian Blue solution was prepared according to manufactured protocol. Slides were first deparaffinised in xylene and re-hydrated through passages in alcoholic solutions and, then, stained in Alcian Blue solution for 30 min. After incubation the staining solution was removed and the culture slides washed to get rid of excessive color. Slides were mounted and examined under light microscope.

### Statistical analysis

Cell count analysis has been performed using Fiji image recognition software.

Data were analyzed as percentage of positive cells on total number of DAPI stained cells. Differences between experimental groups in histological data were evaluated by using two-way ANOVA for culture condition (scaffold in expansion medium, scaffold in chondrogenic medium o pellet differentiation) and timepoint (1, 2, and 4 weeks) for each of the assessed molecular endpoint (Sox9, Matrilin 1, Collagen II, and Aggrecan) followed by Tukey's HSD *post-hoc* test. For all experiments, a *P* < 0.05 was considered significant. All analyses were performed by means of Systat (Systat Software, USA).

## Results

### Scaffold fabrication and characterization

The morphological and microstructural analyses of scaffolds were performed by SEM. The scaffolds images displayed porosity distribution from 16 to 180 μm, with larger channel (excluded from the analysis).

**Figure 1:**
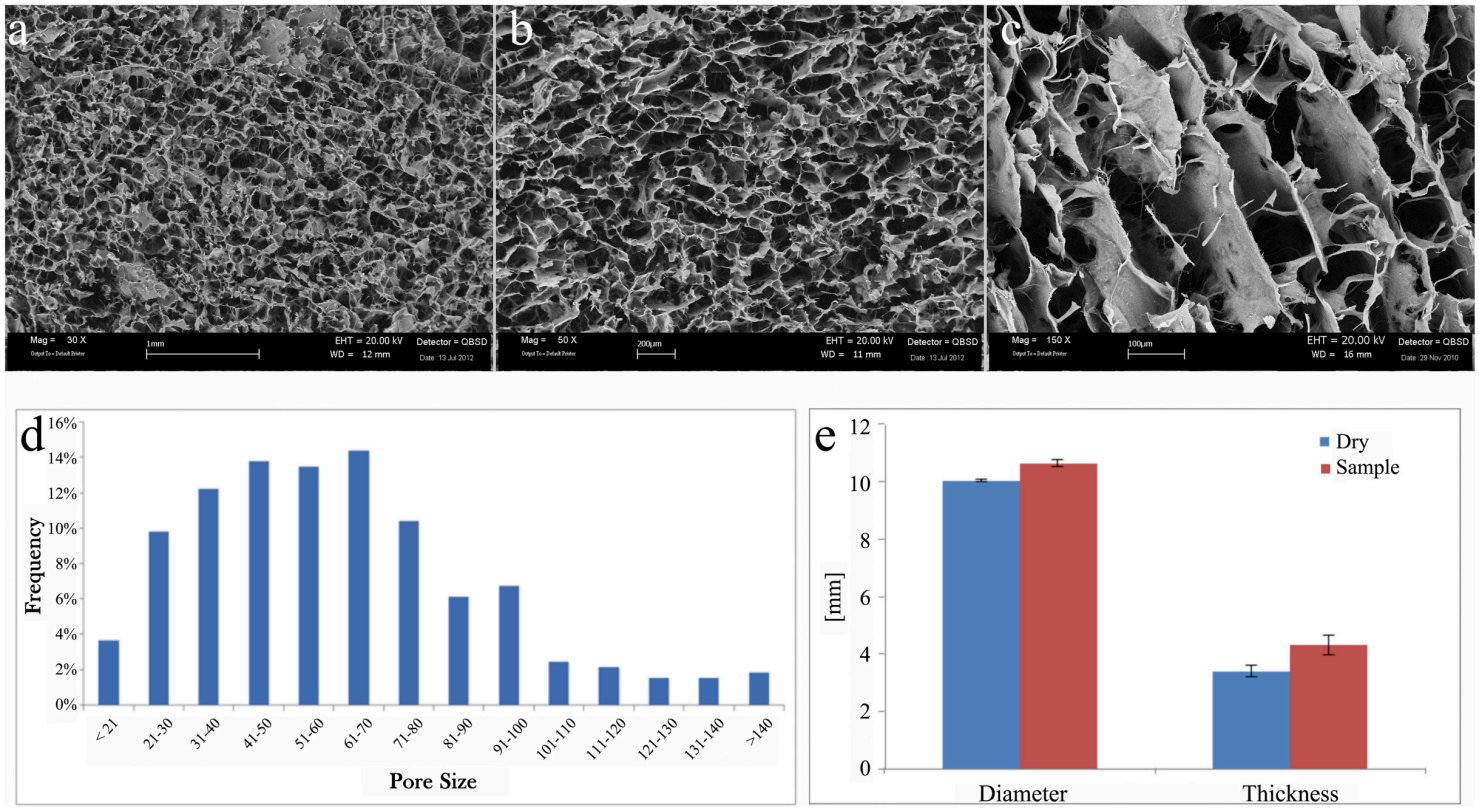
**SEM images of collagen scaffold at (A)** 30x, **(B)** 50x, and **(C)** 150x magnifications. **(D)** Graphical representation of pore size (μm) frequency throughout the scaffold. **(E)** Graphical representation of collagen scaffold swelling behavior relative to the modification of diameter and thickness after soaking in PBS solution. Blue columns, dry samples before soaking; red columns, wet samples after soaking.

SEM images of the cartilage layer are displayed in Figure 1 with different magnification, 30x (Figure 1A), 50x (Figure 1B), and 150x (Figure 1C).

The cartilage-like layer shows a high level of porosity with three-dimensional interconnected pores without any preferential alignment of the collagen fibers. This kind of structures can help the cells function and guide it during their proliferation.

A qualitative analysis of the pores distribution has been performed, and it revealed a higher pores frequency, about the 65%, from 40 to 100 μm (Figure 1D).

Change of material structure was evaluated by swelling test. Each scaffold was weighed before and after the use of PBS solution to soak the scaffold. Swelling test clearly demonstrated that scaffolds are highly hydrophilic reaching the steady state in less than 1 min. The amount of PBS absorbed has been measured to evaluate the collagen scaffold ability to preserve liquid attributed to the maintenance of the three-dimensional structure. The absorption capability of the collagen scaffold resulted 2086 ± 238%.

Both diameter and thickness of the scaffold significantly increased after swelling (Figure 1E). The change in thickness (27 ± 9%) was much higher than in diameter (6 ± 1%).

The porosity of the cartilage in the biomaterial is crucial to assure the cell colonization through the whole scaffold. This parameter is related to the density of the biomaterial forming the scaffolds. The importance of the cell colonization directly influences scaffolds biocompatibility and cell adaptation toward the scaffold. The evaluation of the scaffolds porosity was determined using the medium of the densities obtained through a glass pycnometer and the geometric volume of the cylindrical shaped scaffolds. Applying the reported formula the density found is 0.89 ± 0.22 g/cm^3^ giving a porosity of 95.79 ± 0.29%.

### hADSCs phenotypic characterization

**Figure 2:**
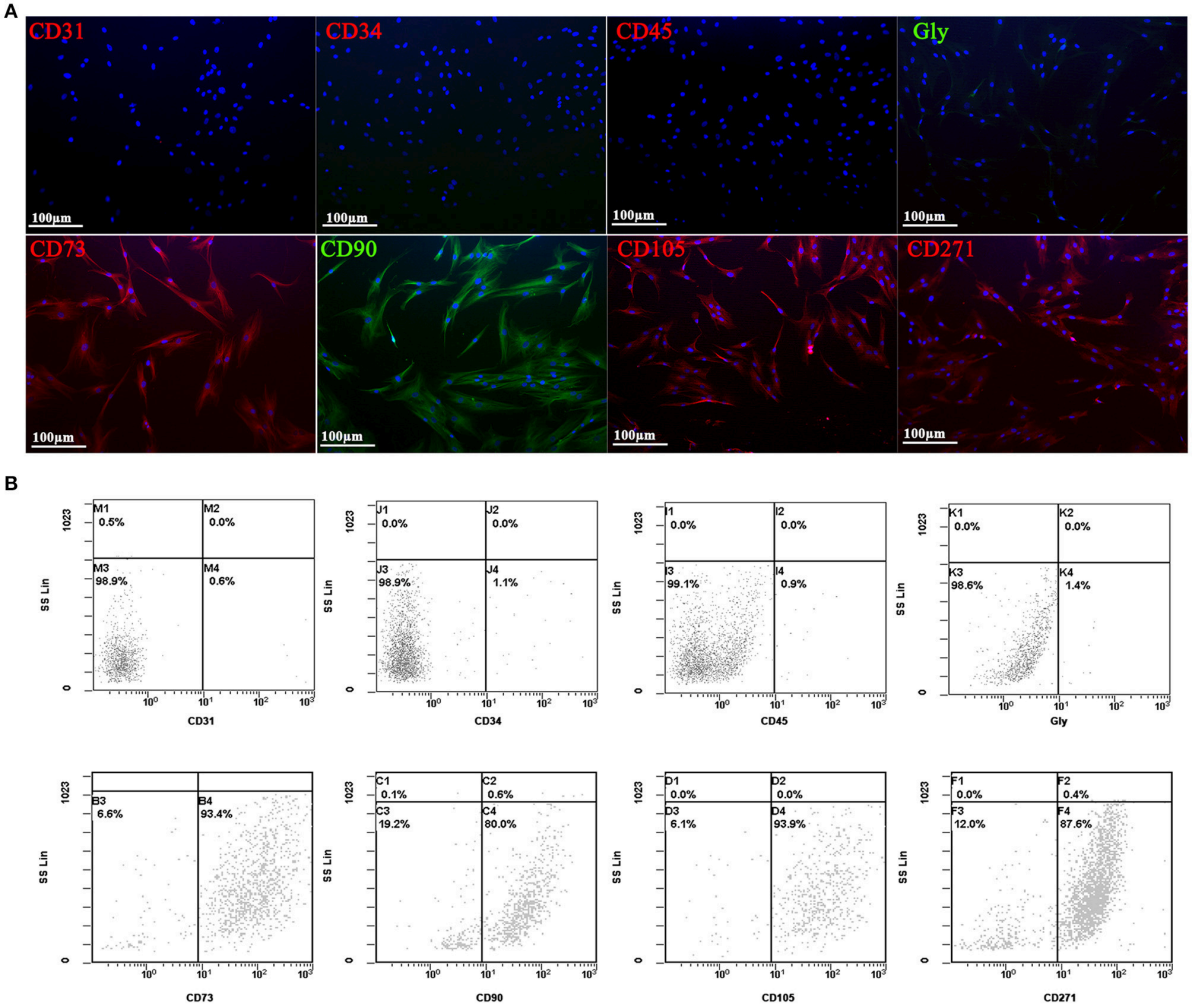
**Immunofluorescent (A)** and flow cytometry analyses **(B)** of negative (CD31, CD34, CD45, and GlycoforinA) and positive (CD73, CD90, CD105, and CD271) mesenchymal stem cells surface markers (red and green stains). Nuclei are labeled in blue. Power magnification: 20x. Scale bar: 100 μm.

All the experiments have been conducted with MSCs isolated from adipose tissue, and characterized by immunocytochemistry (Figure 2A) and flow cytometry (Figure 2B) analysis using several MSCs surface markers. Three different hADSC lines were used to study the expression of typically positive (CD73, CD90, CD105, CD271) and negative (CD31, CD34, CD45, glycoforin A) surface markers of stemness. In both analysis, all cell lines presented a strong positivity for CD73, CD90, CD105, and CD271, while no signal was detected for CD31, CD34, CD45, and glycoforin A (Figure 2).

### Biocompatibility and chondrogenic potential of the collagen scaffold vs. hADSCs

Firstly, we wanted to evaluate the *in vitro* biological performance of a novel 3D porous scaffold, in terms of biocompatibility and ability to support hADSCs to differentiate toward a chondrocyte phenotype.

**Figure 3:**
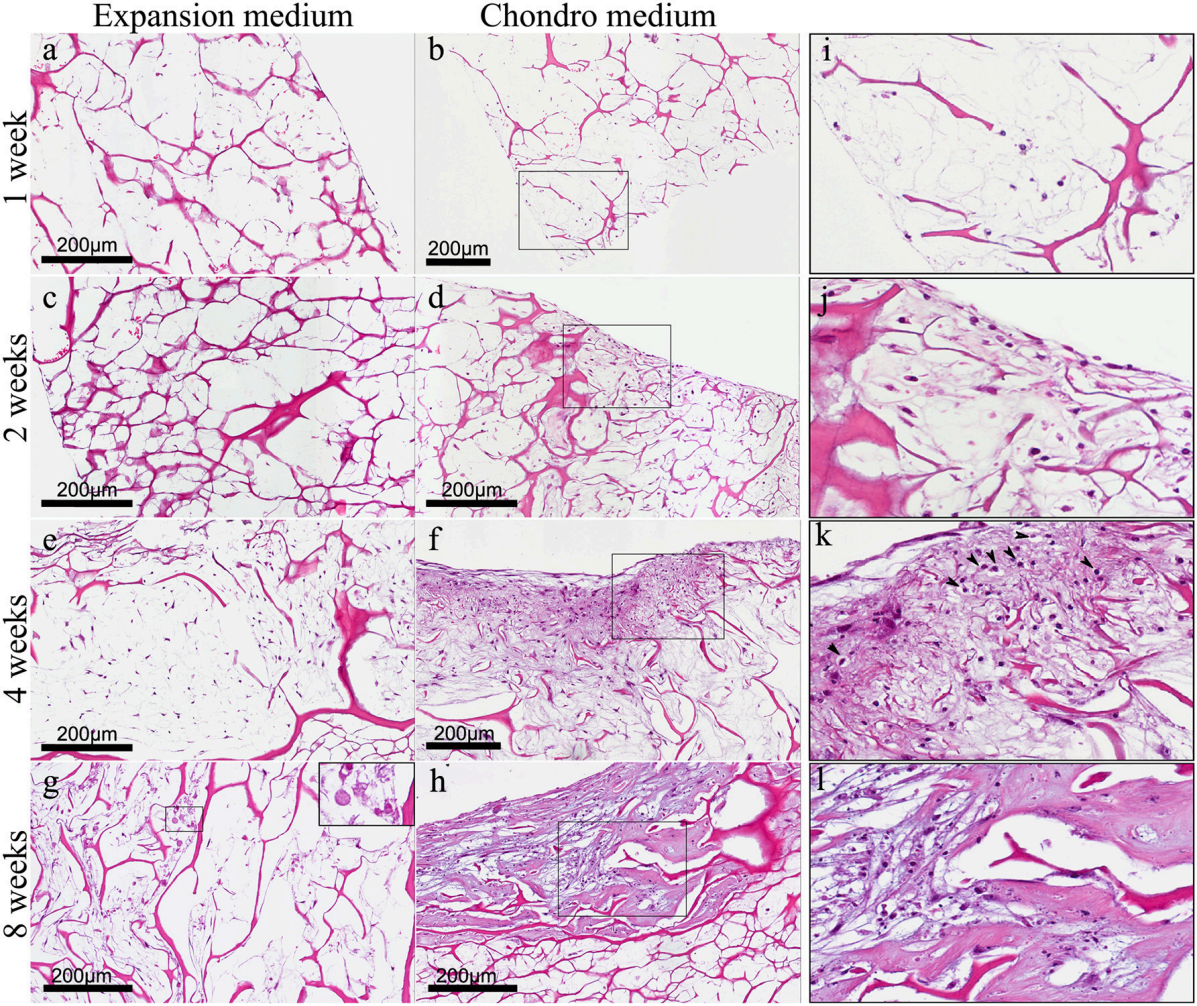
**(A)** Haematoxylin-Eosin staining of hADSC cultured on scaffolds either in absence (expansion medium) or presence (chondro medium) of chondrogenic inducing factors in culture media, after 1 **(A,B)**, 2 **(C,D)**, 4 **(E,F)**, and 8 **(G,H)** weeks of growth in culture, magnification 20x. **(I–L)** higher power magnifications of the squared area, respectively, in **(B,D,F,H)**. The arrows in **(K)** indicate the presence of pericellular lacunae resembling chondrocytes.

We have already demonstrated that hADSCs and bone-like scaffold have a high biocompatibility (Calabrese et al., 2016a). In this work, we found the same high biocompatibility of hADSCs with the collagen-based scaffold, as shown by haematoxylin and eosin (H&E) staining (Figure 3) that reveals the ability of cells to penetrate, adhere and proliferate into the scaffold. In particular, hADSCs loaded onto the scaffold were confined only on the surface during the first 2 weeks (Figures 3A–D), then, cells started to invade also the inner part of the scaffold, penetrating deeply inside (Figures 3E–H). This was observed in both experimental culture conditions: with expansion medium (Figures 3A,C,E,G) and with chondrogenic medium (Figures 3B,D,F,H), although a significant difference between the two experimental conditions was noted, in terms of cellularity and extracellular matrix quality. In fact H&E staining revealed not only the biocompatibility but also the differential proliferative and differentiation potential of the scaffold in the two different experimental conditions. Specifically, in expansion medium, at 1 week, cells are few and still confined on the surface of the scaffold, over the time, from 1 to 4 weeks, cells proliferate and migrate inside the scaffold, the scaffold results wrapped by a layer of spindled to stellate fibroblast-like cells that progressively invade the innermost part of material (Figures 3A,C,E,G). Anyway, at 4 weeks cells begin to appear suffering (Figure 3E), and, surprisingly, cellular necrosis and regressive phenomena occurred at 8 weeks (Figure 3G), as better shown in the squared image at higher magnification (Figure 3G square).

On the other hand, in presence of chondrogenic inducing factors (chondro medium) the increase in cellularity from first to eighth week is much more evident if compared with the same samples without inducing factors (expansion medium). In particular, starting from the second week (Figure 3D) it is also quite evident that the extracellular matrix assumes a chondromyxoid-like feature which progressively increases up to 8 weeks (Figures 3F,H). In fact, MSCs start to go toward condensation and differentiate in chondrocytes. The different nature of the cells is better highlighted in the images at higher magnification (Figures 3I–L) showing spindled and stellate cells during the first 2 weeks (Figures 3I,J). At 4 weeks the presence of pericellular lacunae (arrows) resembling chondrocytes, become quite clear (Figure 3K). At 8 weeks the chondromyxoid-like nature of the extracellular matrix, typical of cartilage tissue, with bigger and more numerous pericellular lacunae, is strongly evident (Figure 3L).

**Figure 4:**
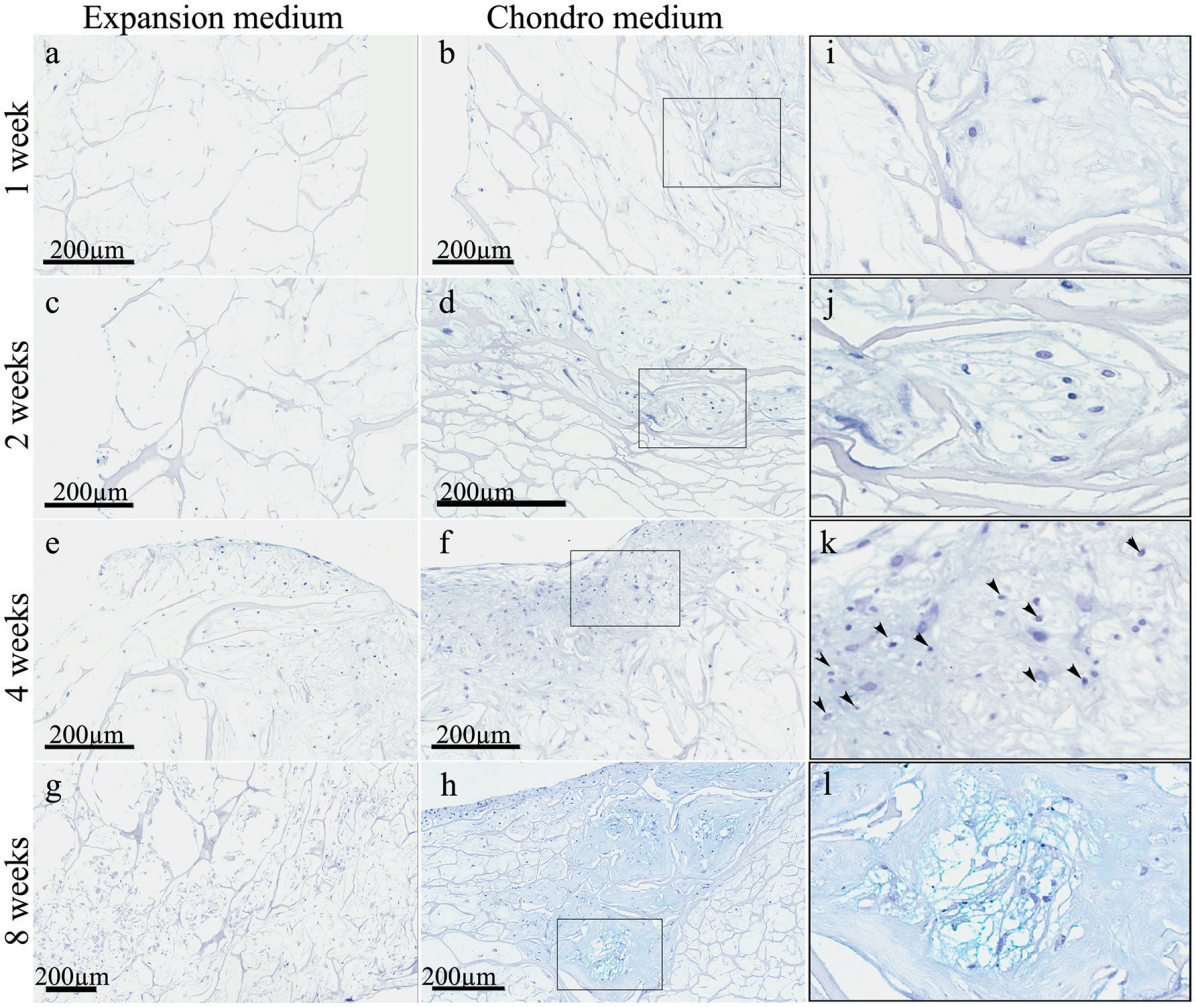
**Alcian Blue staining of hADSC cultured on scaffolds either in absence (expansion medium) or presence (chondro medium) of chondrogenic inducing factors in culture media, after 1 (A,B)**, 2 **(C,D)**, 4 **(E,F)**, and 8 **(G,H)** weeks of growth in culture, magnification 20x. **(I–L)** higher power magnifications of the squared area shown, respectively, in **(B,D,F,H)**. The arrows in **(K)** indicate the presence of pericellular lacunae resembling chondrocytes.

These observations were strongly confirmed by the Alcian Blue staining (Figure 4) that highlighted a striking difference between the samples in expansion medium (Figures 4A,C,E,G) and the samples in chondrogenic medium (Figures 4B,D,F,H), in terms of extracellular matrix nature. In expansion medium the blue staining did not reveal any chondromyxoid-like nature, whereas the presence of bioactive factors in the culture medium lead to the deposition of an extracellular matrix, starting to be visible already at 1 week, in which spindled and stellate mesenchymal cells are still present (Figures 4B,I). From the second week the amount of extracellular matrix deposition is bigger (Figure 4D) and become more and more consistent at 4 (Figure 4F) and 8 weeks (Figure 4H), where chondrocyte-like mesenchymal cells (Figures 4J,K) are embedded in a chondromyxoid-like matrix, presenting the typical cartilage pericellular lacunae, strongly evident in the higher magnification Figure 4L. The intense Alcian Blue staining confirms the cartilage-like nature of the neo-formed tissue.

### hADSCs chondrogenic differentiation performance of scaffold and conditioning medium

In order to assess the chondro-inductive character of the scaffold in combination with the bioactive factors, we decided to compare the performance of the reference protocol defined by the inductive media manufacturer with the ones achievable by culturing of hADSCs on the collagen based 3D scaffold in both absence (expansion medium) or presence (chondro medium) of chondrogenic factors. At this aim we performed immunohistochemical analysis using markers typical of chondrogenic differentiation, including Sox9, type-II Collagen, Aggrecan and Matrilin-1.

**Figure 5:**
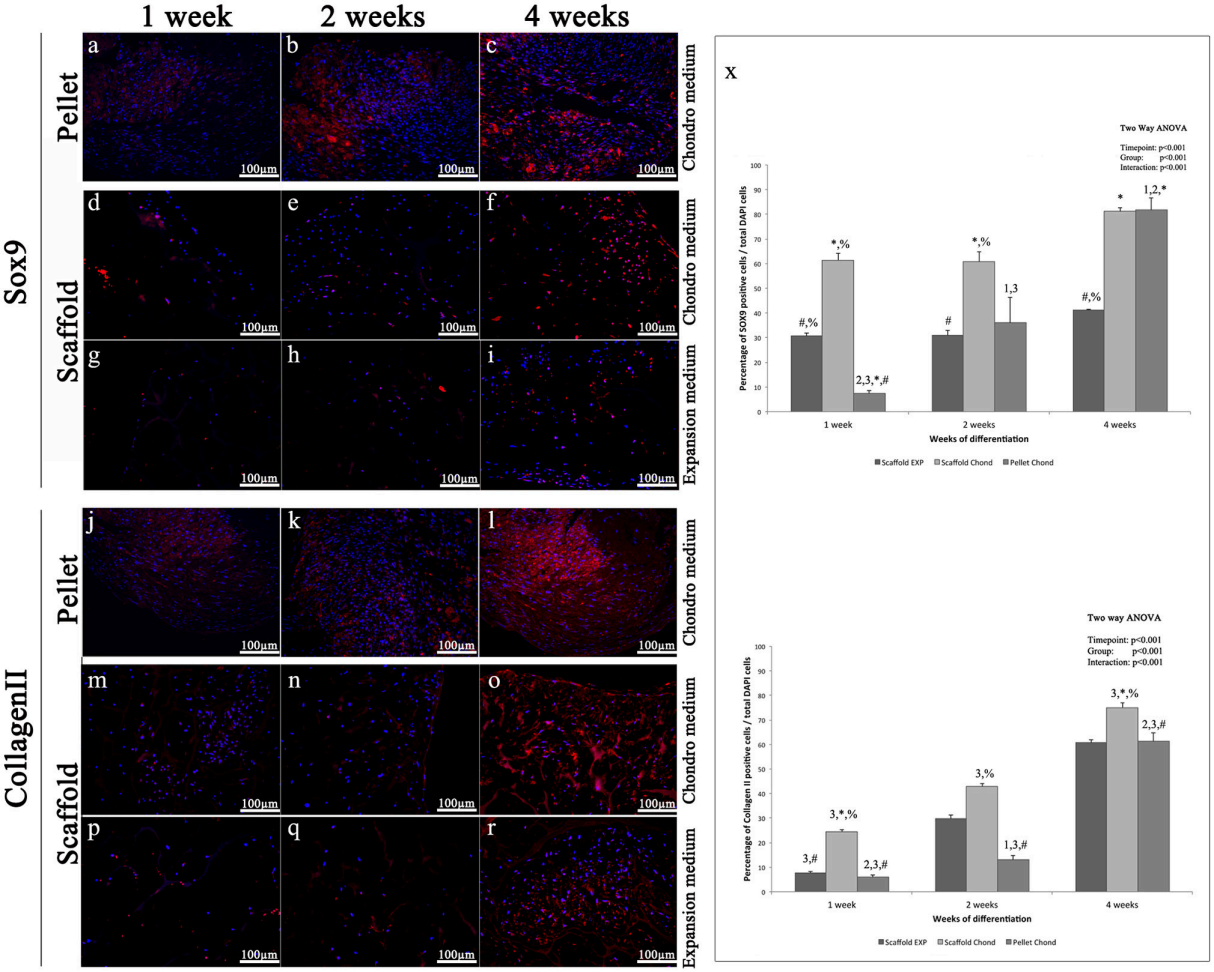
**Immunofluorescent analysis of representative chondrogenic markers: (A–I)** Sox9, **(J–R)** type II Collagen, performed on hADSCs and hADSCs cultured on scaffolds either in expansion **(G–I,P–R)** or in chondrogenic **(A–F,J–O)** medium, at different time points (1, 2, and 4 weeks), magnification 20x. **(X)** Average cellular positivity for chondrogenic markers in both expansion (dark bars) and chondrogenic medium (dark and medium bars). Percentage of Sox9 and Collagen II **(X)** are calculated on the total number of DAPI stained cells in the investigated fields. Two-way ANOVA p values are reported. Symbols above bars indicate statistically significant differences (*p* < 0.05) in the Tukey HSD *post-hoc* tests: 1 indicates differences with the 1 week group (same medium), 2 indicates differences with the 2 weeks group (same medium), 3 indicates differences with the 4 weeks group (same medium), 4 indicates differences with the 8 weeks group (same medium), ^*^ indicates differences with scaffold expansion medium group (same time-points), # indicates differences with scaffold chondrogenic medium group (same timepoints) and % indicates differences with pellet group (same timepoints). Average cellular positivity for chondrogenic markers at 2 (dark bars) and 4 weeks (light bars). Three independently isolated hADSC samples have been used for each time-point.

Sox9 is a transcription factors that controls the expression of type II Collagen and Aggrecan and has a crucial role in chondrogenesis (Bi et al., 1999). The immunohistochemical analysis of Sox9 shows only few labeled nuclei during the first week in all experimental conditions (Figures 5A,D,G), at the second weeks a more evident fluorescent signals is present only in the pellet (Figures 5B,E,H), at 4 weeks a further increase is clearly visible in the pellet (Figure 5C), while the scaffold samples show a slight augment of Sox9 marked nuclei in both conditions, although in chondro medium (Figure 5F) such increase results higher compared to the expansion medium (Figure 5I).

In accordance with Sox9, Type-II Collagen immunohistochemical analysis shows a very weak staining during the first two weeks in all three conditions (Figures 5J,K,M,N,P,Q), although slightly more evident in the pellet (Figures 5J,K), whereas, starting from 4 weeks a more robust and extended signal becomes visible, although it results significantly higher in the pellet and in the scaffold samples in presence of inducing factors (Figures 5L,O), compared to the scaffold without bioactive factors (Figure 5R).

**Figure 6:**
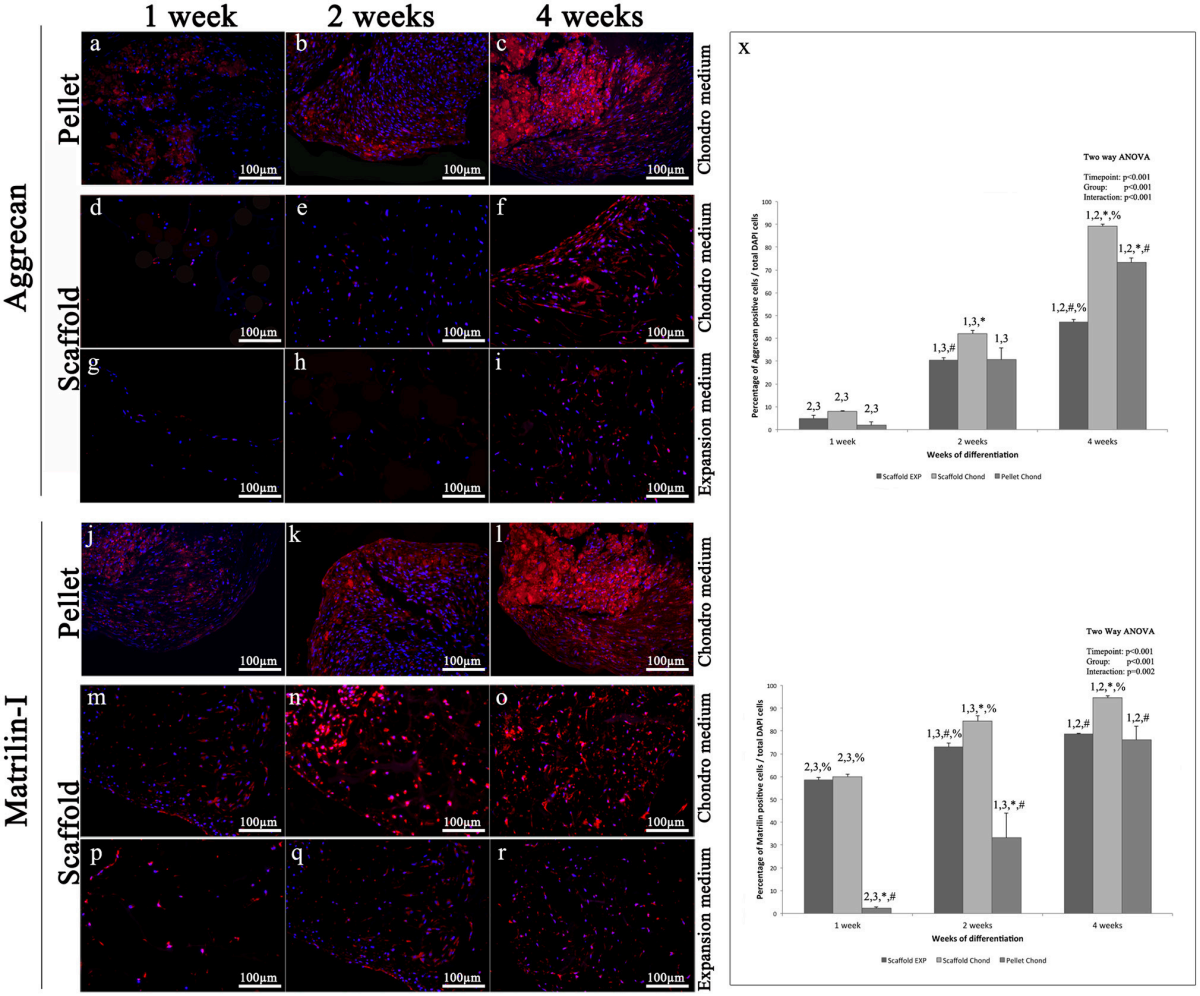
**Immunofluorescent analysis of representative chondrogenic markers: (A–I)** Aggrecan, **(J–R)** Matrilin, performed on hADSCs and hADSCs cultured on scaffolds either in expansion **(G–I,P–R)** or in chondrogenic **(A–F,J–O)** medium, at different time points (1, 2, and 4 weeks), magnification 20x. **(X)** Average cellular positivity for chondrogenic markers in both expansion (dark bars) and chondrogenic medium (dark and medium bars). Percentage of Aggrecan and Matrilin **(X)** are calculated on the total number of DAPI stained cells in the investigated fields. Two-way ANOVA p values are reported. Symbols above bars indicate statistically significant differences (*p* < 0.05) in the Tukey HSD *post-hoc* tests: 1 indicates differences with the 1 week group (same medium), 2 indicates differences with the 2 weeks group (same medium), 3 indicates differences with the 4 weeks group (same medium), 4 indicates differences with the 8 weeks group (same medium), ^*^ indicates differences with scaffold expansion medium group (same time-points), # indicates differences with scaffold chondrogenic medium group (same timepoints) and % indicates differences with pellet group (same timepoints). Average cellular positivity for chondrogenic markers at 2 (dark bars) and 4 weeks (light bars). Three independently isolated hADSC samples have been used for each time-point.

Aggrecan immunohistochemical analysis shows a protein expression pattern comparable to that of Type-II Collagen. Specifically, immunohistochemistry for Aggrecan reveals almost no signal during the first 2 weeks in both experimental conditions of the scaffold samples (Figures 6D,E,G,H), while in the pellet is already present a visible fluorescence at 1 week (Figure 6A) that becomes a little higher a 2 weeks (Figure 6B); at 4 weeks the pellet fluorescent staining is very strong (Figure 6C). At 4 weeks in scaffold, a modest fluorescent labeling is present in expansion medium (Figure 6I), while a quite stronger staining appears in chondrogenic medium (Figure 6F). Aggrecan is the principal proteoglycan (GAG) present in the ECM and forms, along with type-II collagen, the main structural element of the cartilage. As far as type-II Collagen, Aggrecan at 4 weeks presents a filamentous appearance (Figures 6C,F,I).

Matrilin-1 results very weakly expressed during the first 4 weeks of scaffold cultures in absence of differentiation inducing factors (Figures 6P–R). Conversely, in presence of chondrogenic inducing factors, already at 1 week a modest signal was visible (Figures 6L,O) both in pellet and scaffold samples (Figures 6J,M). Over time, the intensity and the extent of the fluorescent staining gradually increases until to be very strong at 4 weeks (Figures 6K,L,N,O), even if always a little higher in the pellet.

Results indicate that statistically significant differences in markers expression exist between all the differentiation methods assessed (*p* < 0.001), as graphically presented in Figures 5X, 6X.

## Discussion

During life, hyaline cartilage of articular joints is constantly exposed to several static and dynamic compression loads due to both normal activities, such as stair climbing (Hodge et al., 1986), and traumatic events. Such continuous stresses lead to a progressive degeneration of the cartilage with consequent pain, swelling, loss of function and, finally, osteoarthritis (or degenerative joint disease) that represents the most common musculoskeletal disorder (Jackson et al., 2001).

Still, cartilage healing remains a challenge mainly because of its avascular nature. Several approaches aimed to restore injured cartilage, have been pursued by researchers and clinicians. Among these, cell-based strategies have provided some encouraging clinical results (O'Driscoll, 1998; Richardson et al., 1999; Wakitani et al., 2011; Jo et al., 2014), although, the major drawback of such approach is the failure in generating a three-dimensional tissue having characteristics similar to native cartilage concerning quality and stability (Vinatier et al., 2009a). Newer tissue engineering strategies are focusing in developing new chondro-inductive biomaterials capable of inducing a complete healing of cartilage lesions in combination with chondrogenic bioactive growth factors.

While various studies have shown the capability of MSCs derived from different tissues to generate cartilage (Nakahara et al., 1990; Arufe et al., 2010; Oldershaw, 2012), bone marrow and adipose tissue are considered the most attractive sources for therapeutic use of MSCs in cartilage repair and regeneration (Somoza et al., 2014). However, bone marrow-derived MSCs have an intrinsic endochondral ossification potential, definitely higher than that of ADSCs (Mehlhorn et al., 2006; Diekman et al., 2010), leading to the development of a hypertrophic chondrocyte phenotype (Johnstone et al., 1998; Scotti et al., 2010), which is not appropriate for articular cartilage repair.

Recently, it has been reported that MSCs expressing CD105, CD29 (Rada et al., 2011; Fan et al., 2016), as well as CD271 (Calabrese et al., 2015) revealed a greater chondrogenic potential compared to other MSC subpopulations, including those derived from bone marrow (Calabrese et al., 2015). Moreover, CD271-positive MSCs exhibited the highest level of type II collagen and Aggrecan, after chondrogenic induction (Nakahara et al., 1990).

Here we propose a novel combination of a collagen based 3D scaffold, CD271-positive MSCs, isolated from adipose tissue (hADSC) and chondrogenic differentiation inducing soluble factors as a potential tool for cartilage regeneration.

Several types of scaffold, both of synthetic and natural origin, have been already reported in literature as suitable tool for cartilage engineering (Vinatier et al., 2007, 2009b; Zeugolis et al., 2008; Eglin et al., 2010; Solorio et al., 2013; Dahlin et al., 2014; Tsai et al., 2015; He et al., 2016; Hung et al., 2016; Raftery et al., 2016). A scaffold is a three-dimensional structure capable to support cell colonization, proliferation, and differentiation of appropriate cells. Stimuli mimicking the *in vivo* cartilage environment are needed for pushing cells to differentiate into mature chondrocytes.

We have recently demonstrated that a collagen-hydroxyapatite scaffold is able to commit human MSCs toward osteogenic differentiation, *in vitro* (Calabrese et al., 2016a) and *in vivo* (Calabrese et al., 2016b). Accordingly, in this study, we have evaluated the ability of a novel scaffold composition, to induce hADSCs to differentiate into chondrocytes, *in vitro* and compared the differentiation potential of hADSCs seeded on scaffold and in cellular pellet.

Chondrogenic differentiation of condensed cellular pellet is defined as a standard protocol for the *in vitro* commitment of MSC toward chondrogenic lineage using the above specified conditioning medium (see Materials and Methods section). The chondrogenic potential of this procedure has been used as benchmark to evaluate differentiation potential of hADSC cultured in collagen based scaffold both in presence and absence of the conditioning medium. The chondrogenic differentiation was evaluated by immunofluorescent analysis of specific cartilage markers. In particular, (i) Sox9, a transcription factor playing a crucial role in chondrogenesis, which controls the expression of type II Collagen and Aggrecan; (ii) type-II Collagen, that is the core of articular and hyaline cartilage, it forms fibrils and represent more than 50% of all protein and about the unique type of collagen in articular cartilage; (iii) Aggrecan, that forms, together with type-II Collagen, a major structural component of cartilage, and represents the major proteoglycan in the articular cartilage; (iv) Matrilin-1, which is a protein involved in the formation of filamentous networks in the cartilage ECM interacting with both type-II Collagen and Aggrecan in the ECM. All the markers used have shown their highest expression at the last time point studied of hADSCs chondrogenic differentiation (4 weeks). Results indicate that statistically significant differences in markers expression exist between all the differentiation methods assessed (*p* < 0.001). In particular is clearly evident that when hADSC are cultured on scaffold the percentage of SOX9 and Matrilin positive cells is significantly higher after 1 week of differentiation both in presence or absence of soluble factors. When conditioning factor are not present the augmented expression of chondral markers observed in scaffold cultured cells decrease with time, with cells differentiated in pellet showing a comparable (Collagen II and Matrilin-1) or significantly augmented (SOX9 and Matrilin-1) protein expression. In almost every time point investigated, cells cultured on 3D scaffold in presence of conditioning medium exhibit a significantly higher expression of all the chondral markers observed suggesting that hADSC are committed faster than in the other culturing conditions.

In our model, the scaffold is composed principally by type-I Collagen. Importantly, the scaffold biomaterial has shown a high biocompatibility, defined as the ability to support host cell proliferation and differentiation and to promote the formation of the extracellular matrix on scaffold surface and pores.

The data obtained from our *in vitro* studies have shown that the scaffold, *per se*, seemed to be sufficient to induce differentiation of hADSCs layered on top into early chondrocyte precursors. This has been demonstrated by the appearance of lineage specific markers, although in less extent and in later time compared to the samples grown in chondrogenic medium. On the other hand, H&E and Alcian Blue stainings have shown not only the absence of a chondromyxoid-like ECM in expansion medium, but also that at 4 weeks cells became suffering and underwent to cellular necrosis and regressive phenomena. Hence, our hypothesis is that, in absence of bioactive factors, the scaffold can promote an initial hADSCs differentiation into early chondrocyte precursors, which begin to express chondrogenic markers; but, afterwards, the scaffold alone is not able to drive a complete chondrogenic differentiation, leading cells to death. On the other hand, the addition of chondro-inductive factors to the culture medium (chondro medium samples) significantly speeded up the differentiation process, as suggested by the stronger and earlier chondrogenic marker expression. Moreover, the H&E and Alcian blue stainings revealed not only an increase in cellularity but also the production of an ECM having a chondromyxoid-like nature. Moreover, cells embedded in the ECM are surrounded by pericellular lacunae, strongly resembling chondrocytes of mature cartilage.

In conclusion, the data presented in this work demonstrated that hADSCs could represent a source of election of MSCs to use in cartilage tissue engineering applications, due to their easy harvest and chondrogenic potential (Calabrese et al., 2015). Moreover, the collagen scaffold used showed a high biocompatibility with hADSCs. The *in vitro* data showed that the biomaterial composing the scaffold possesses the intrinsic property of promoting an initial chondrogenic differentiation of hADSCs, even if not sufficient to lead to the complete hADSC differentiation into chondrocytes. However, the combination of scaffold with bioactive factors strongly accelerates the process leading to the formation of a new-formed tissue strongly resembling cartilage.

Hence, the present work suggests that the combination of hADSCs, collagen biomaterial and chondrogenic inducing factors, could represent a promising tool to be used in tissue engineering for restoration and regeneration of damaged articular cartilage.

## Ethics statement

This study was carried out in accordance with the recommendations of “Good Clinical Practice (GCP) of European Community” with written informed consent from all subjects. All subjects gave written informed consent in accordance with the Declaration of Helsinki. The protocol was approved by the “IOM Institutional Review Board” with protocol code: 829_1 of 8 February 2013.

## Author contributions

All authors had substantial contribution to the present work. Specifically, GC conceived the project, designed and performed the experiments; TM performed the quantification of the immunohistochemistry experiments; SF and RoG executed the statistical analysis and have been involved in revising the manuscript critically for important intellectual content, SF is also the corresponding author; FC and EF supplied the scaffolds and contributed to their construction and characterization and participated in manuscript writing; LM supplied the surgical sample for the isolation of MSCs and have done H.E. staining; GA and LS have done histology, microscopy and have participate to data analysis; RP and MG participated to project preparation, data interpretation and manuscript writing; RaG conceived the project, designed and performed the experiments, and wrote the paper, she is also the corresponding author. All the authors gave the final approval of the version to be published.

## Funding

The present research was partially funded by the Italian “PON Ricerca e Competitività 2007–2013, ASSE I 829” grant program entitled “Piattaforme tecnologiche innovative per l'ingegneria tissutale.”

### Conflict of interest statement

The authors declare that the research was conducted in the absence of any commercial or financial relationships that could be construed as a potential conflict of interest.

## Abbreviations

MSCs: Mesenchymal Stem Cells
ECM: Extracellular Matrix
hADSCs: Human Adipose Derived Stem Cells
DAPI: 4′,6-diamidino-2-phenylindole
GAG: Glycosaminoglycans
H&E: Haematoxylin and Eosin
